# Comparative Three‐Dimensional Morphology of Baleen: Cross‐Sectional Profiles and Volume Measurements Using CT Images

**DOI:** 10.1002/ar.23648

**Published:** 2017-10-03

**Authors:** Megan M. Jensen, Amalia H. Saladrigas, Jeremy A. Goldbogen

**Affiliations:** ^1^ Hopkins Marine Station of Stanford University Pacific Grove California 93950

**Keywords:** baleen, CT scanning, morphology, mysticetes, hydrodynamics

## Abstract

Baleen whales are obligate filter feeders, straining prey‐laden seawater through racks of keratinized baleen plates. Despite the importance of baleen to the ecology and natural history of these animals, relatively little work has been done on baleen morphology, particularly with regard to the three‐dimensional morphology and structure of baleen. We used computed tomography (CT) scanning to take 3D images of six baleen specimens representing five species, including three complete racks. With these images, we described the three‐dimensional shape of the baleen plates using cross‐sectional profiles from within the gum tissue to the tip of the plates. We also measured the percentage of each specimen that was composed of either keratinized plate material or was void space between baleen plates, and thus available for seawater flow. Baleen plates have a complex three‐dimensional structure with curvature that varies across the anterior‐posterior, proximal‐distal, and medial‐lateral (lingual‐labial) axes. These curvatures also vary with location along the baleen rack, and between species. Cross‐sectional profiles resemble backwards‐facing airfoils, and some specimens display S‐shaped, or reflexed, camber. Within a baleen specimen, the intra‐baleen void volume correlates with the average bristle diameter for a species, suggesting that essentially, thinner plates (with more space between them for flow) have thinner bristles. Both plate curvature and the relative proportions of plate and void volumes are likely to have implications for the mechanics of mysticete filtration, and future studies are needed to determine the particular functions of these morphological characters. Anat Rec, 300:1942–1952, 2017. © 2017 The Authors The Anatomical Record published by Wiley Periodicals, Inc. on behalf of American Association of Anatomists

## INTRODUCTION

Baleen whales (Mysticeti) are obligate filter feeders that include the largest animals that have ever lived on Earth. Despite reaching massive body sizes, these animals filter‐feed on aggregations of small‐bodied organisms, such as krill, copepods, and fish, by straining prey‐laden seawater through baleen. Baleen consists of a series of keratin laminae, arranged transversely in two racks that erupt from the gums on either side of the animal's upper jaw (Williamson, 1973; Werth, 2000, Fig. 1A). The number of plates per rack varies by species, ranging from 100 to 400 laminae (e.g., Nemoto, 1970; Pivorunas, 1979; Werth, 2000). Plate length also varies by species, from approximately 50 cm to 5 m, with plates spaced approximately 1 cm apart (Werth, 2000); however, plate spacing may vary slightly along the length of the rack (Young, 2012).

**Figure 1:**
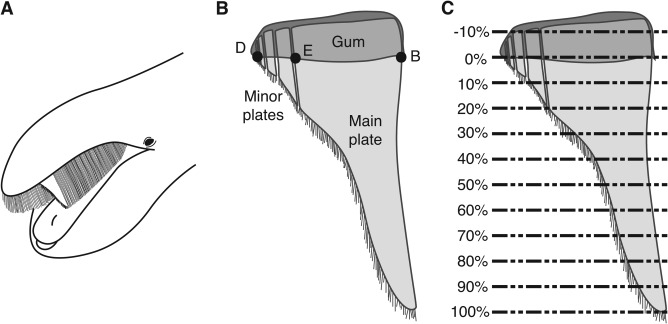
Baleen morphology. (A) Cutaway of baleen rack of a generalized mysticete, showing a baleen plate (redrawn from Werth, 2013, with permission). (B) A baleen lamina, showing points B, E, and D from Williamson (1973). Points B and D were used to define the proximal end of a baleen plate (redrawn and modified from Williamson, 1973). (C) Baleen lamina from B), showing the proximal (0%) and distal (100%) end of the plate, as well as the 11 locations where cross‐section images were made (‐10% to 90%).

Baleen plates are composed of keratin tubules embedded in a keratinized matrix (e.g., Pivorunas, 1979; Werth, 2000). Plates are worn down over time, so baleen grows continuously over the animals’ lifetime. The tissue from which the plates grow is known as the Zwischensubstanz, which provides spacing between plates and stress dissipation for plates subjected to large forces from feeding (Tullberg, 1883 in Fudge et al., 2009; Pinto and Shadwick, 2013). It is thought that constant wear causes baleen plates to fray on the interior edge, creating a fibrous mat of bristles (Pivorunas, 1976). These bristles, also called baleen fringes, reorient in flow (Werth, 2013), though the exact role of these bristles in the filtration process remains unclear.

The extant baleen whales consist of four families that exhibit largely divergent filter feeding modes (Werth, 2007). The bowhead and right whales, of family *Balaenidae*, use continuous ram filtration and skim‐feed at the surface (Werth, 2004; Simon et al., 2009). In contrast, members of *Balaenopteridae*, or rorquals (e.g., humpback, fin, blue, sei, and minke whales), use intermittent lunge feeding to engulf seawater (Simon et al., 2012; Goldbogen et al., 2017). The gray whale is the only extant member of *Eschrichtiidae*, and it suction feeds on organisms from benthic sediment (Johnson and Nelson, 1984; Oliver and Slattery, 1985). A fourth family, *Neobalaenidae*, consists only of the pygmy right whale, and very little is known about its feeding mechanism beyond inferences based on the shared morphological characteristics with balaenids (Kemper, 2009; Marx and Fordyce, 2016). Baleen morphology reflects the different feeding modes used by the balaenids, rorquals, and gray whales: first, balaenid baleen plates are much longer than those of the rorquals or gray whale, reaching lengths of 4–5 m in the bowhead whales. In contrast, rorqual baleen plates reach a maximum length of 1 m. Second, balaenid plates are much narrower, with finer bristles than rorqual or gray whale plates. In balaenids, small bristle diameters correspond with small prey size; balaenid whales filter feed on copepods and other small (<10 mm) zooplankton (Nemoto, 1970; Werth, 2000). Gray whales possess short plates and thick, coarse, and stiff bristles, which may facilitate the filtration of sediment‐ridden water from the benthos (Young, 2012; Jones et al., 2012; Young et al., 2015).

The hydrodynamic mechanisms by which baleen filters seawater remain unknown, though recent work by Werth and Potvin (2016) and Potvin and Werth (2017) has illuminated the likely filtration process used by balaenids. Their work suggests that right and bowhead whales use cross‐flow filtration rather than sieve, or throughput, filtration. Thus, rather than the baleen bristles and plates acting as a colander to strain prey from the water, most of the flow moves along the anterior‐posterior axis of the animal. A small amount of seawater exits between each plate as flow moves along the anterior‐posterior axis, creating a slurry of prey in the mouth that grows more concentrated as it moves toward the esophagus. This filtration method has the distinct advantages of both eliminating the need for the whale to remove prey from its baleen (no definitive method for removing prey from baleen has been identified, (Werth, 2001)), and preventing the filter from clogging with prey items. Cross‐flow filtration (CFF) and vortical cross‐step filtration (Sanderson et al., 2016) have also been identified in bony fishes (Cheer et al., 2001; Brainerd, 2001; Sanderson et al., 2001) and elasmobranchs (Paig‐Tran et al., 2013; Paig‐Tran and Summers, 2014). Balaenid whales feature both morphological and behavioral adaptations that create the necessary pressure gradients to enable and support CFF during the continuous forward motion of the animal while feeding (Werth and Potvin, 2016; Potvin and Werth, 2017).

In contrast to the continuous filter‐feeding methods employed by balaenids, rorqual whales intake large volumes of prey‐laden water in a single gulp, then expel that water through the baleen (Werth, 2000). The process is repeated, often several times, at depth during a foraging dive, and thus has been classified as an intermittent filter feeding mechanism (Goldbogen et al., 2017). The morphology of balaenopterid baleen plates reflects the differences between lunge feeding and continuous ram feeding: rorqual baleen plates are much shorter and have wider bristles than those of the right and bowhead whales, and they tend to be much wider at the base (Werth, 2000). Balaenopterids are typically assumed to use sieve filtration, although cross‐flow filtration has been hypothesized as a potential mechanism based on a simple model of flow through baleen in fin whales (Goldbogen et al., 2007). However, the precise filtration mechanism(s) used by rorquals remains unknown and requires further investigation.

Despite the importance of baleen to the ecology of mysticetes, only a few studies have characterized the morphology of baleen. This is due in large part to logistical difficulties in obtaining these tissues. Williamson (1973) defined several measurements of baleen racks, plates, and laminae, including width, length, bristle diameter, plate spacing, and bristle density. These markers were used by Young (2012) and Young et al. (2015) to describe baleen morphology for several specimens across 10 species. The cross‐sectional profiles of baleen plates are also of interest; presumably these shapes have hydrodynamic effects on the flow through plates. Cross‐sectional profiles of baleen have been described by Scoresby (1811), Pivorunas (1976), and Lambertsen et al. (2005). The plate curvature is presumed to stiffen baleen plates and prevent deformation under the hydrodynamic forces produced by expelled water (Pivorunas, 1976; Lambertsen et al., 2005). Lambertsen et al. (2005) also noted that the curvature of balaenid plates, which varies both from the proximal to distal ends as well as along the anterior‐posterior axis, likely contributes to maintaining the alignment and integrity of the filtration apparatus. Lambertsen et al. (2005) also speculated that plate curvature might produce a Venturi effect that increases flow rates through plates.

Our understanding of how baleen functions is far from complete, and is limited at least partially by disparate studies of baleen morphology that preclude direct comparison. Baleen racks are three‐dimensional structures with curvature along the anterior‐posterior, dorsal‐ventral, and medial‐lateral (lingual‐labial) axes. However, most published records of baleen morphology simplify the various measurements to two dimensions. While these measurements have been correlated with various ecological and evolutionary traits (prey type, feeding strategies, phylogenetic signals, Young, 2012), characters describing the three‐dimensional structure have not been made. Further, beyond the descriptions of Pivorunas (1976) and Lambertsen et al. (2005), no quantitative descriptions of baleen plate cross‐sections and their variations among mysticete families and species have been carried out.

To answer these questions, we used computed tomography (CT) scanning to image baleen specimens from both museum collections and stranded animals to describe the 3D structure of baleen. We imaged cross‐sectional profiles of baleen plates at multiple locations along the anterior‐posterior axes and distal‐proximal axes to describe the varying cross‐sectional shapes among mysticetes. We also quantified the percentage of each specimen (by volume) that is comprised of either plate material or void between baleen plates (intra‐baleen, or IB void), and thus space through which seawater might flow. We then correlated these measurements with known 2D morphological characters and prey preferences.

## METHODS AND MATERIALS

### Obtaining and Imaging Specimens

Baleen was obtained from two museum specimens and four stranded animals (Table 1). Two specimens from the marine mammal collections at the Smithsonian Institution National Museum of Natural History were imaged: one apparently complete rack (left side) from a sei whale (*Balaenoptera borealis)* calf (USNM 593931), and a complete rack (both left and right sides) from a minke whale (*Balaenoptera acutorostrata*) calf (USNM 593554). Partial racks collected from stranded animals included two fin whales (*Balaenoptera physalus*): one adult that stranded in British Columbia, Canada (RR 2015–0127), and a sub‐adult that stranded in California (TMMC‐C‐473). Partial baleen racks also came from an adult blue whale (*Balaenoptera musculus*) that stranded in Oregon (HMSC15–11‐01‐Bm), and a gray whale (*Eschrichtius robustus)* calf that stranded in California (TMMC‐C‐477). Domestic stranded specimens were stored under permit to MMJ by the National Oceanic and Atmospheric Administration's National Marine Fisheries Service.

**Table 1:** Museum and stranded baleen specimens used in this study

Species	ID (museum or stranding number)	Animal length (m)	Animal age class	Stranding Date	Scanning Date	Number of plates measured	Cross‐section analysis	Baleen sample location along rack
Blue whale *B. musculus*	HMSC15–11‐01‐Bm	21.3	Adult^a^	Nov 2015	Nov 2015	18	No	Mid‐anterior Right side
						14	Yes	Mid‐anterior Right side
Fin whale *B. physalus*	TMMC‐C‐473	15.9	Subadult^a^	Aug 2015	Nov 2015	37	Yes	Anterior Left side
	RR 2015–0127	13.0	Adult^a^	May 2015	Jun 2015	37	No	Mid‐anterior Left side
Sei whale *B. borealis*	USNM 593931	6.7	Calf (likely unweaned)^b^	Mar 2014	Aug 2015	37	No	Mid‐anterior Left side
						37	Yes	Middle Left side
						N/A	Yes	Mid‐posterior Left side
						37	No	Posterior Left side
Minke whale *B. acutorostrata*	USNM 593554	3.2	Calf (likely unweaned)^c^	Feb 2012	Nov 2015	20	No	Middle Left side
						20	No	Posterior Left side
Gray whale *E. robustus*	TMMC‐C‐477	6.6	Juvenile^a^ Calf (likely unweaned)^d^	Sep 2015	Nov 2015	37	No	Middle Right side
						N/A	Yes	Mid‐anterior Right side
						N/A	Yes	Mid‐posterior Right side

a Per stranding report.

b Lockyer (1977).

c Lockyer (1981), Masaki (1979), Williamson (1975), Ohsumi (1979).

d Sumich (1986).

Frozen baleen specimens were CT scanned at one of three machines. The stranded fin whale baleen specimen from Vancouver was scanned at the CT Imaging Centre at FPInnovations Wood Products Division in Vancouver, BC, Canada, using an industrial 4‐MeV scanner with a 1‐m scanning area, with CT slice images taken at 1 mm intervals and 0.4 mm resolution. The sei whale and minke whale baleen museum specimens were scanned at the Smithsonian Institution National Museum of Natural History using a medical Siemens SOMATOM Emotion 6 CT scanner, with images with a resolution of 0.72 mm taken at 0.3 mm intervals. The gray whale, humpback whale, and California fin whale specimens were imaged at the Community Hospital of the Monterey Peninsula with a GE Healthcare Lightspeed Pro 16 scanner, with slices imaged at 0.625 mm intervals with approximately 1 mm resolution (A 3D animation of representative CT data is shown in Supplementary Video 1: https://players.brightcove.net/656326989001/default_default/index.html?videoId=5529242263001).

### Plate Cross‐Section Measurements

Images of cross‐sections of three plates for six samples from four specimens were obtained using ImageJ (Rasband, 1997, U.S. National Institutes of Health, Bethesda, MD). Cross‐sections were imaged for the mid‐anterior blue whale baleen sample, the California fin whale baleen sample, and two locations from both the gray whale (mid‐anterior and mid‐posterior) and sei whale (middle and mid‐posterior) baleen racks. Williamson (1973) described cross‐sections of a baleen lamina using three points: B, at the lateral, outer‐most edge; E, at the medial edge of the main plate, and D, at the medial edge of the entire lamina (including minor plates, Fig. 1B). We imaged lamina cross‐sections for three contiguous plates of each specimen at 11 locations parallel to the BD axis described by Williamson along the length of the lamina. With the BD axis representing the proximal end of the plate (0% of the plate length) and the distal tip of the main plate representing 100% of the plate length, we imaged plate sections at 10% length intervals from 0%, to 90%, as well as one measurement within the Zwischensubstanz at −10% (Fig. 1C). Because specimens were not scanned with consistent orientations, image stacks for each specimen were first rotated and resliced to produce image stacks that were oriented similarly and parallel to the BD axis.

### Plate and IB Void Volume Measurements

To measure relative volumes of baleen material and intra‐baleen (IB) space, representative sections varying from 20 to 37 plates were chosen from the six baleen specimens. The blue whale baleen samples were two adjacent pieces from the same rack, and were analyzed separately. The complete racks of the USNM sei and minke whale baleen specimens allowed us to measure 37‐plate sections from the anterior, middle, and posterior portions of the sei whale rack, and two adjacent 20‐plate sections from one side of the minke whale rack. Because the minke whale rack was small, these adjacent sections encompassed the middle and posterior regions of the rack. Samples were chosen to maximize the number of complete plates captured by the scanner (the BC fin whale, blue whale, and minke whale samples) or to match the 37 plates measured from the Vancouver fin whale, the first sample imaged (CA fin whale, gray whale, and sei whale samples). In total, 10 data points were measured from 6 specimens representing 5 species.

**Figure 2:**
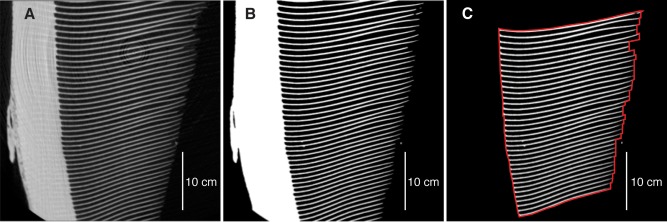
Steps used to calculate plate, IB void, and total volumes in ImageJ. (A) Original CT image. (B) Thresholded image, eliminating grayscale values. (C) Cropped image that includes only complete plates, and removed Zwischensubstanz and bristle clumps. Pixels in white represent the area composed of plate material, and the red outline represents the boundary for measuring total area. IB void area is calculated by subtracting plate area from total area.

The images acquired with the CT scans were arrayed in an image stack of baleen sample cross sections using ImageJ. Because the specimens were scanned with various orientations on the scanner bed, CT image stacks were first re‐oriented so images showed baleen plates in the plane created by the proximal‐distal and anterior‐posterior axes (Fig. 2A). We used threshold tools in ImageJ to remove gray shades from the images to enable the automatic measuring tools in the software (Fig. 2B). (Comparisons of measurements using user‐defined plate areas on nonthresholded images and thresholded images suggest thresholding may increase plate areas up to 18%; this is explored in the Discussion). Finally, we cropped the image stacks to include only complete baleen plates that were not truncated by the CT scanner, and removed broken plates. For each image, we defined plate areas by removing both the Zwischensubstanz and any material between plates assumed to be clustered bristles or foreign material (Fig. 2C); generally, bristles were not dense enough to appear in the CT images unless they were tightly clustered. We measured the area representing plate material in each edited image using automatic area measurement tools in ImageJ (white pixels in Fig. 2C). To measure the IB void for each image, we first measured the total area of each image bounded by the edge of all plates (red outline in Fig. 2C), then subtracted the area of the previously‐measured plate material (shown in white). These measurements were repeated for images at approximately 5 mm intervals along the medial‐lateral axis. Due to the small size of the minke whale baleen specimen, plate areas and IB spacing were measured at images spaced at intervals of approximately 2 mm.

Plate volume, intra‐baleen void volume, and total volume (plate plus IB void) were calculated by integrating the areas measured for each of these parameters across the thickness of the plates at the image spacing intervals (5 mm or 2 mm). Lastly, we normalized plate and intra‐baleen volumes in all specimens by the total volume to facilitate comparisons between specimens.

## RESULTS

### Cross‐Sectional Profiles

**Figure 3:**
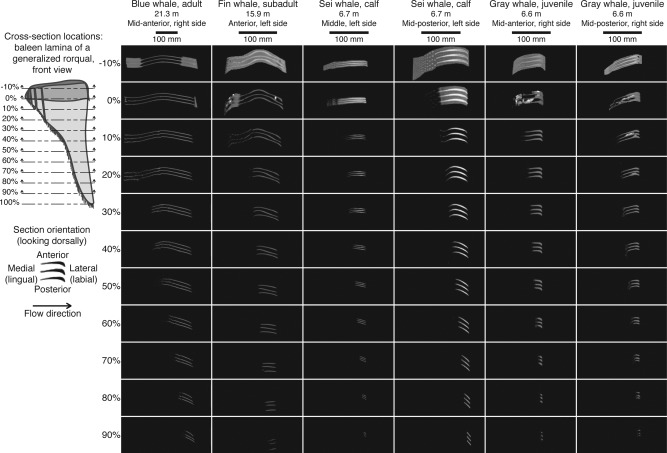
Cross‐sectional profiles at 11 locations along the length of three baleen laminae.

Several general patterns were evident in the cross‐sectional profiles in Figure 3. In general, the plates curved such that the lateral/labial edges pointed in the posterior direction (as noted by Pivorunas (1976) and Lambertsen et al. (2005)). In many of the specimens, certain plates were cracked; such cracks are visible in the blue whale section cuts between 50% and 80%. In all six samples, the plate spans tapered along the proximal‐distal axis; specifically, the plates became narrower toward the tips, seemingly due to wear. The curvature in the plates appears to flatten toward the distal end—proximal sections (e.g., 10%–20%) generally have more spanwise curvature than the distal ones (e.g., 60%–90%). This is likely an artifact of the plates eroding at the medial edges along the length of the plate; curvature at the lateral edges of the plates remains quite consistent along the entire plate length. Many of the cross‐sectional profiles, particularly those from the gray and sei whales, resemble those of airfoils, with one critical difference: airfoils have the maximum thickness near the leading edge, while the baleen cross‐sections are widest near the trailing edge. Thus, the cross‐sections presented in Figure 3 resemble airflows in a reversed flow direction.

The plates comprising the blue whale specimen displayed “reflex camber;” that is, the curvature along the three plates changed direction. The medial‐most portions of the profile were concave anteriorly, while the lateral sides of the sections were concave posteriorly. As the plate narrowed along its length, the lateral‐side curvature was preserved, and the medial‐side curvature disappeared between the 20% and 30% sections. The maximum curvature occurred at the approximate midpoint between the medial and lateral edges in the −10% to 20% sections.

**Figure 4:**
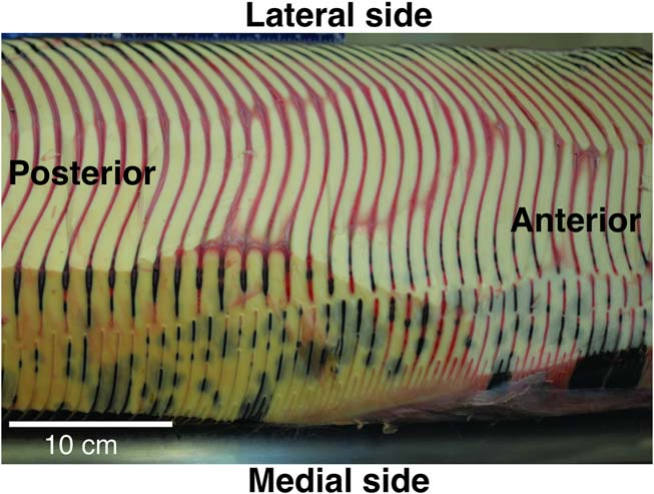
Photograph of Zwischensubstanz from Canadian fin whale, showing both posterior divergence (top of image, plates curve to left) and reflex camber (present in plates on right side of image).

The fin whale specimen also had reflex camber in the −10% and 0% sections. Though not included in the cross‐section analysis, the Canadian stranded fin whale specimen also showed reflex camber in the plates visible through the Zwischensubstanz (Fig. 4), demonstrating the double curvature is not unique to the California individual. The fin whale curvature was strongest in the middle of the plate (halfway between the medial and lateral edges) at the −10% and 0% sections. From the 10% sections on along the length of the plate, the fin whale specimen plate flattened rapidly; the sections from 60% to 90% were very nearly flat. In addition, there was twisting toward the posterior direction at the lateral edges in the fin whale relative to the other five specimens.

The gray whale specimen had foreign matter (primarily mud) stuck in many of the plates along the length of the rack; this is visible in the 0% sections for both gray whale columns in Figure 3 and the 10% section for the posterior gray whale specimen. This mud somewhat obscured the plate shapes of the 0% sections. While for most specimens the bristles were not dense enough to appear clearly on CT images, the coarse bristles of the gray whale are visible in several sections, and appear as “clumps,” particularly in the posterior specimen. The maximum thickness of the gray whale plates was located at the lateral edge, and this thickness tended to be greater than that of the blue, fin, and sei whale plates at the same section location. The curvature was strongest at the point of maximum thickness; since this occurred at the lateral edge, this curvature was preserved as the plate narrowed. As a result, the gray whale specimens were more curved at the distal sections than the blue, fin, and sei whale plates.

The plates from the middle of the sei whale rack were the least curved of all six sets of cross‐sections. All sections of the plates were essentially flat, excepting the slight curvature at the lateral edge of the −10% and 0% sections. The thickness along the width of the plate was relatively uniform in the plates from the middle of the rack, while the plates from the posterior of the rack were much thicker at the lateral side of the proximal end (‐10% and 0% sections). Plates from the posterior of the rack had close to a uniform thickness from the 10% section through the 90% sections, though they were more curved than the plates from the middle of the same rack. The 70%–90% sections appeared essentially flat. Plates from the posterior of the rack curved toward the posterior at the lateral edge more prominently than do the plates from the middle of the rack, and were also longer in length and thicker at comparable sections.

### Plate/IB Volume Measurements

The total intra‐baleen void volume, plate volume, and overall volume varied according to the species and age of each whale, as well as the size of the sample (Table 2). The anterior measurement of the sei whale rack showed the largest normalized intra‐baleen void volume; the middle and posterior measurements showed a gradual decrease in IB void volume. The gray whale sample had the smallest normalized IB void volume, and consequently, the largest plate volume, which is consistent with the thicker plates and bristles of these bottom‐feeders. The minke whale had the second‐largest measurements of normalized IB void volume. The Canadian fin whale sample had a smaller IB void volume than either of the blue whale baleen measurements, while the fin whale sample from California had a greater IB void volume.

**Table 2:** Normalized plate, intra‐baleen (IB) void, and total volumes

Species	Baleen sample location along rack	Number of plates measured	Total IB void volume (m^3^)	Total plate volume (m^3^)	Overall volume (m^3^)	Normalized plate volume	Normalized IB void volume
Blue whale *B. musculus*	Mid‐anterior	18	5.44E‐03	1.76E‐03	7.20E‐03	0.244	0.756
	Mid‐anterior	14	5.89E‐03	1.93E‐03	7.82E‐03	0.246	0.754
Fin whale *B. physalus*	Anterior	37	4.03E‐03	1.16E‐03	5.20E‐03	0.224	0.776
	Mid‐anterior	37	6.73E‐03	2.39E‐03	9.12E‐03	0.262	0.738
Sei whale *B. borealis*	Mid‐anterior	37	2.40E‐04	4.69E‐05	2.83E‐04	0.165	0.835
	Middle	37	4.79E‐04	1.11E‐04	5.90E‐04	0.188	0.812
	Posterior	37	6.74E‐04	1.84E‐04	8.58E‐04	0.214	0.786
Minke whale *B. acutorostrata*	Middle	20	4.10E‐05	1.00E‐05	5.10E‐05	0.196	0.804
	Posterior	20	2.72E‐05	7.69E‐06	3.49E‐05	0.220	0.780
Gray whale *E. robustus*	Mid‐anterior	37	5.93E‐04	3.10E‐04	9.03E‐04	0.343	0.657

Although we have too few data points for any statistical significance, our data may suggest a trend toward decreasing IB void volume in more posterior baleen locations (Table 2). In both the sei and minke whales, there is an increase in plate volume and corresponding decrease in IB void volume along the anterior‐posterior axis. The blue whale samples, which were directly adjacent on the same individual's rack, had nearly identical IB void volumes, though the more posterior measurement was slightly smaller. Though the two fin whale samples are from separate animals, the more anterior section of baleen had a greater normalized IB void volume.

## DISCUSSION

This study presents the first three‐dimensional morphology measurements of baleen using CT scanning, which were previously impossible to make nondestructively. In this study, we quantified the cross‐sectional profiles of baleen plates along the proximal to distal ends. We also looked at the relative volume of baleen plates vs the intra‐baleen (IB) voids between plates; this void space is presumably available for seawater flowing through baleen laminae during filtration.

### Cross‐Sectional Profiles

The cross‐sectional profiles of rorqual baleen plates have not attracted significant research attention, despite the likelihood that these shapes are very important to baleen filtration flow patterns. One of the most visually apparent results from Figure 3 is how similar plates in profile resemble conventional airfoils: this is especially true for the gray whale and sei whale specimens. One might speculate that these sectional shapes have evolved to achieve hydrodynamic benefits in flow, but this neglects the flow direction: in conventional airfoils, the point of maximum thickness is located at the leading edge of the wing, and the airfoil tapers gradually toward the trailing edge. For baleen plates, the leading edge is the medial edge, while the maximum thickness location is at the lateral, or trailing edge, opposite of a conventional airfoil. Airfoil profiles are designed to maximize lift while minimizing drag, and thus reduce the thrust necessary to move them through a fluid (e.g., Anderson, 1985). Though minimizing drag during filtration would have an energetic advantage for mysticetes (possibly enabling large body sizes, Potvin and Werth, 2017), the hydrodynamic advantages, if any, from the “reversed” airfoil profiles are unknown without measuring fluid dynamic forces exerted on baleen plates. It is likely that this shape offers a compromise between optimizing flow through plates (e.g., drag reduction or filtration efficiency) and structural stability, but experimental tests are needed to confirm this hypothesis. An additional complication to the flow between plates is that the medial edges of the main plates are not the first part of the filtering apparatus that engulfed seawater encounters, at least at the proximal end of the plates: seawater exiting the mouth must first pass through rows of minor plates and bristles frequently arranged in complicated patterns. Flowing around these features will have a hydrodynamic cost in the form of increased drag; the as‐yet‐unknown functional roles of the bristles and minor plates may thus confer advantages that outweigh this cost. Flow tests are also needed to determine the relative magnitudes of lift and drag produced by individual plates as seawater flows through baleen laminae.

Several of the specimens analyzed—the blue whale, California fin whale, and a photo of the Canadian fin whale specimen (Fig. 4), show cross‐sections with “reflex camber,” or double curvature. This shape was first noted in baleen by Scoresby (1811), and described by Pivorunas (1976) as a “sinuous course to the slitlike spaces that pass between consecutive spaces” that offers structural rigidity to the plates, particularly in the center. In airfoils, reflex camber (resembling an “S” rather than a “C” shape) decreases the amount of overall lift generated by an airfoil, as the upswept trailing edge provides negative lift, which minimizes the torque about the center of the airfoil (e.g., Abbott and Doenhoff, 1959; Pope, 2009). In effect, wings with reflex camber act as their own horizontal stabilizer, and can be used in experimental aircraft without horizontal stabilizers (e.g., Kroo, 1993). Determining any hydrodynamic and/or structural advantages produced by reflex camber in baleen filtration will require flow tank studies, but we can speculate possible benefits here. Any curvature in the plate is likely to offer some structural support, and stiffen the plate, as noted by Pivorunas (1976) and Lambertsen et al. (2005). In all of our specimens, the degree of curvature was greatest at or within the gum (‐10% and 0% sections), where the plates are anchored. This is not unexpected: for cantilevered beams, maximal bending stresses occur at the beam's base. For baleen plates subject to bending forces during filtration, curvature‐induced stiffness would therefore be most effective where the plates erupt from the Zwischensubstanz, which has stress‐dissipating material properties (Pinto and Shadwick, 2013). Increasing plate stiffness to resist bending stresses may also explain why plate cross‐sections are thickest at the proximal ends within the gum tissue.

Posterior curvature at the lateral edges of the plates was noted by Lambertsen et al. (2005) in balaenid baleen; this was described as unique to the balaenids and absent from rorquals. However, all the specimens we observed displayed this feature to varying degrees (Figs. 3 and 4), and Pivorunas (1976) showed a figure of a sei whale plate in cross‐section with this posterior curvature. Lambertsen et al. (2005) hypothesized that this posterior divergence was a method of keeping the long (up to 4 m) balaenid baleen plates in alignment during filtration, and that it might contribute to faster flows through plates due to a Venturi effect. Our results are consistent with that hypothesis, as many of the images in Figure 3 appear to show plates that are closer at the lateral edge than in the middle or medial edge. However, this effect cannot be quantified without hydrodynamic studies. For rorquals in particular, the triangular shape of baleen plates and narrowing width of the plate along their length does not necessarily suggest that flow moves transversely through the plates with no dorsal‐ventral component. The three‐dimensional streamlines taken by seawater through the plates are likely too complicated to simplistically model as pipe flow, and thus we cannot assume that plates narrowing in a 2D slice lead to a Venturi effect between plates without directly testing this hypothesis experimentally.

Our cross‐section descriptions may help describe the 3D flows between baleen plates in the future by better informing hydrodynamic models used in computer simulations of baleen flow. Additionally, by quantifying the curvature along the proximal‐distal, medial‐lateral, and anterior‐posterior axes, we might build more accurate models of filtration in mysticetes in three dimensions, improving on those such as the 2D model in Potvin and Werth (2017). These data might also be used to make physical models of baleen plates for flow tank studies.

### Baleen Morphology, Prey Preferences, and Void Volume

**Figure 5:**
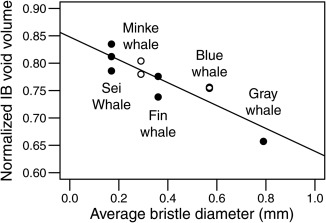
Normalized intra‐baleen (IB) void volume vs. average bristle diameter for the ten volume measurements. IB void volume decreases with average bristle diameter (linear least‐squares regression, *P* < 0.001).

Void volume was significantly correlated with average bristle diameter (Fig. 5, linear least‐squares regression, *P* < 0.001). This is not unexpected, as void volume increases with decreasing plate volumes. Thus, thinner plates were associated with more void space and finer bristles. Gray whales, which are benthic suction‐feeders, have the thickest bristles of the species measured, suited to withstand sediment filtration and strong influxes of water (Young, 2012 and Young et al., 2015); they also had the smallest measured IB void volume. Minke and sei whale baleen plates have the smallest bristle diameter, and our results showed these specimens have the greatest IB void volume. Sei whales have bristle diameters that are very similar to the fine bristles seen in balaenids, likely due to their similar feeding preferences targeting small copepods and using skim feeding (Kawamura, 1974; Young, 2012). We also tested relationships between IB void volume and prey size for each measured specimen using the data in Table 3. We found no correlation between plate or IB void volumes and average prey size, nor any statistically significant relationships between plate/void volumes and either actual animal length or the maximum length reached by each species (linear least‐squares regression, *P* > 0.5 in all cases). However, these data are from different species that are not equally related, and no phylogenetic corrections were made, as that is beyond the scope of this article. In the meantime, our results should be interpreted with the appropriate caution, and phylogenetic comparative analyses should be used on a larger data set in the future.

**Table 3:** Animal sizes, average bristle diameters, and typical prey items/sizes

Species	Animal length (m)	Maximum adult size (m)	Average bristle diameter^c^, ^d^(mm)	Preferred prey items^c^, ^d^, ^e^	Typical prey size (mm)
Blue whale *B. musculus*	21.3	33^a^	0.57	Krill	10–45
Fin whale *B. physalus*	15.9	27^a^	0.36	Krill Small schooling fish	10–45 250–450
	13.0				
Sei whale *B. borealis*	6.7	20^a^	0.17	Copepods Krill	4–5.5 10–45
Minke whale *B. acutorostrata*	3.2	11^a^	0.29	Small fish Krill	250–450 10–45
Gray whale *E. robustus*	6.6	15^b^	0.79	Mysids Amphipods	8–9 10–15

a Klinowska (1991).

b Reeves and Mitchell (1988).

c Nemoto (1959).

d Young (2012).

e Nemoto (1970).

### Location along A/P Axis

For the blue, minke, and sei whale baleen specimens, we had specimens large enough to make plate/void volume measurements at 2–3 locations along the rack, and we found a decrease in normalized intra‐baleen void volume along the anterior‐posterior axis (Table 2). Though we do not have enough data points for each species for this trend to be statistically significant, we can speculate on the hydrodynamic implications of this trend if it were shown to be statistically and biologically valid across specimens and species.

Previous studies based on assessments of baleen wear (Werth et al., 2016b) and two‐dimensional measurements of baleen morphology (Young, 2012) indicate that there may be increased water flow through the posterior section of the rack. In Young's study of the comparative morphology of baleen across 10 species of mysticetes, Young also identified an increase in plate spacing and thicker, sturdier plates at the posterior of the rack in a full, dried rack of minke whale baleen. Werth et al. (2016b) observed patterns in baleen wear across eight mysticete species, and noted that reoriented fringes tend to occur at the posterior end of the rack, suggesting greater flow rates posteriorly. At first glance, our results might appear to contradict the observations by Young (2012) and Werth et al. (2016b), as larger flow rates would likely suggest greater IB void volumes to accommodate those flow rates. However, there are two possible explanations for this apparent contradiction. First, Young (2012) observed thicker plates at the posterior end of the rack, which is consistent with our own cross‐sectional results—the sei whale specimen had thicker baleen plates in the posterior location (Fig. 3). If the plates are thick enough, the volume between plates may be reduced despite an increase in plate spacing. Second, rather than an increase in flow volume in the posterior region of the rack, flow velocities might be increased due to increased plate thickness and resultant constriction between plates. This is consistent with observations by Werth and Potvin (2016) and Potvin and Werth (2017) for balaenid filtration, in which flows through baleen plates were found to be the fastest at the posterior end of the rack. If future measurements support our observations of decreasing IB volume posteriorly, and this trend were to be both true and statistically significant, the thicker plates and constricted flows through the posterior portion of the rack might suggest a mechanism by which posterior plates show more wear, and possible evidence for cross‐flow filtration in rorquals. However, as discussed above, we cannot assume a Venturi effect from constricting IB voids without testing this phenomenon in a flow tank.

### Specimen and CT Scanning Limitations

The physical and mechanical properties of baleen change with hydration state (Werth et al., 2016a). Although we sought to use specimens that were as fresh as possible or well‐preserved, we were limited by the availability of specimens. The length of time specimens were exposed to air between collection and scanning ranged from a matter of days for the blue whale to over 3.5 years for the minke whale (Table 1). Though all samples were frozen in the interim periods, some warping due to drying may be present in our data and our results should be interpreted with some caution—particularly for samples with long times between specimen collection and scanning. However, effects from warping are unlikely to affect our volume measurements, as plate material is not added or lost. This might have caused artificial twisting or bending of the plates in cross‐section, particularly at the distal end of the plates. Nevertheless, we predict any error from warping is likely to be slight, given the relatively small amount of twisting displayed in Figure 3. Additionally, the minke whale, which had the longest air exposure, was not included in the cross‐section analysis.

Three of our baleen specimens—the gray whale, minke whale, and sei whale—came from calves small enough that they were unlikely to be weaned at the time of baleen collection. It is probable that baleen from these individuals was never used to filter seawater. Very little, if any, data exist regarding ontological changes to baleen morphology. Given that baleen continually grows throughout the animal's lifetime, it seems reasonable to assume that baleen grows isometrically with the animal. Thus, we assume that baleen from an unweaned calf has the same morphological characters described here as juvenile and adult baleen. Though limiting our work to adult baleen would have been optimal, baleen specimens are very limited.

Though it allows us to make measurements that were previously impossible, CT scanning is not without limitations, particularly in analyzing the images. Images were frequently ambiguous as to where a plate ended and a cluster of bristles began. For the plate/IB void volume measurements, we dealt with this ambiguity as conservatively and consistently as possible; we only removed artifacts that were clearly extraneous to the plates. However, attempting to minimize subjectivity in our measurements introduced an additional degree of error: by thresholding images to measure plate and void areas, plates were artificially thickened by the software. We estimated this error to be up to 18%. Because thresholding was consistently applied to all images, all volumes are likely to be equally over‐ or underestimated, even though plate volumes may be overestimated (and IB void volumes consequently underestimated). Thus, the relationships between volumes and morphological characters, rack location, and feeding preferences are therefore not affected by errors resulting from image thresholding.

In summary, CT scanning enabled us to study the three‐dimensional characteristics of baleen in a way that had previously been impossible without destroying valuable museum specimens or intact specimens from stranded animals. In the future, the three‐dimensional structures we have imaged can be used to directly test the functional roles of morphological characters of baleen. Additionally, these results will help build more accurate hydrodynamic models for computer simulations of mysticete filtration, further illuminating the interplay of morphology and mechanics required for mysticete filtration.

## Supporting information


**This article includes AR WOW Videos. Video 1 can be viewed at** https://players.brightcove.net/656326989001/default_default/index.html?videoId=5529242263001 **.**

Video 1. Animation showing 360‐degree rotation of three‐dimensional representation of partial rack of sei whale baleen, generated from CT images. Baleen plates are from the middle of the rack on the left side of the animal, at the approximate location of the plates used for both cross‐sectional profile analyses and volume calculations.
